# Visual detection of collective highly toxic metals in water by a handheld whole-cell biosensing detector: From circuit to field

**DOI:** 10.1016/j.isci.2026.115289

**Published:** 2026-03-10

**Authors:** Shanshan Pi, Liang Feng, Qiongzhang Wang, Wenjun Yang, Shanshan Yu, Zhao Li, Xiang Gao, Lu Lu

**Affiliations:** 1 State Key Laboratory of Urban-rural Water Resource and Environment, School of Ecology and Environment, Harbin Institute of Technology, Shenzhen 518055, China; 2 State Key Laboratory of Quantitative Synthetic Biology, Shenzhen Institute of Synthetic Biology, Shenzhen Institutes of Advanced Technology, Chinese Academic of Science, Shenzhen 518000, China; 3 Faculty of Synthetic Biology, Shenzhen University of Advanced Technology, Shenzhen 518000, China; 4 State Key Laboratory of Optoelectronic Materials and Devices, Institute of Semiconductors, Chinese Academy of Sciences, Beijing 100083, China

**Keywords:** Chemistry, Engineering, Materials science

## Abstract

Whole-cell biosensors can detect toxic aquatic heavy metals without requiring expensive instruments and trained operators, making them promising for on-site detection. However, most remain confined to laboratory research, with few developed into integrated devices, and face challenges in simultaneously quantifying two highly toxic heavy metals, cadmium and mercury. To address this, we developed a portable platform for the simultaneous detection of them in contaminated water. We first engineered *Vibrio natriegens* into a whole-cell fluorescent biosensor. Paired with this biosensor, we built a palm-sized, battery-operated fluorescent reader featuring temperature-controlled incubation. This reader converts visual signals directly into numerical readings without computer processing. The biosensor can tolerate total metal concentrations up to 5 mg L^−1^ and collectively quantify Cd^2+^ and Hg^2+^ as low as 10−100 μg L^−1^ in real river water samples. At ∼$401.5 per device and ∼$0.33 per test, it is more cost-effective than commercial field detection devices or commercial kits.

## Highlights

• We designed a biosensor that tolerates toxic metal concentrations up to 5 mg L^−1^
• We developed a portable platform for detecting highly toxic metals in water
• We established a direct pathway to bridge biosensor design and device development

## Graphical abstract

**Figure:**
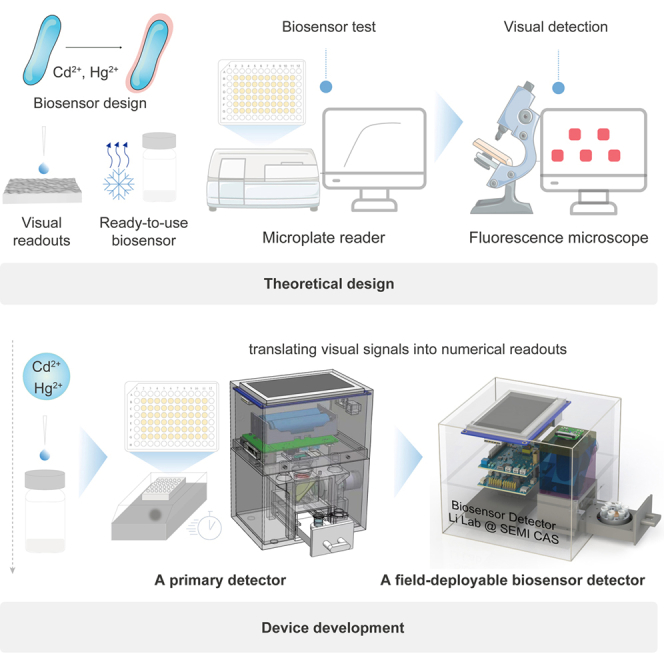

## Introduction

Whole-cell biosensors have been widely used for monitoring heavy metals in water, particularly their bioavailable level, as target metals must traverse the cell membrane to interact with living cells.^1^^,^^2^^,^^3^^,^^4^ Compared with conventional methods such as inductively coupled plasma mass spectrometry (ICP-MS) and atomic absorption spectroscopy (AAS), whole-cell biosensors show great potential to eliminate the need for centralized and expensive instrumentation, trained laboratory personnel, and complex sample pretreatment.^5^^,^^6^^,^^7^ However, most current efforts have focused on improving laboratory biosensor metrics, such as sensitivity and specificity,^8^^,^^9^^,^^10^ while the transition from theoretical designs to field-deployable devices for non-specialists remains challenging, particularly for highly toxic analytes. Two major bottlenecks hinder practical application. First, contaminated waters often contain mixtures of highly toxic heavy metals, such as cadmium (Cd) and mercury (Hg). Both are priority pollutants where even trace levels can cause irreversible kidney and nervous system damage and increase cancer risk. Thus, simultaneous detection is essential. However, developing whole-cell biosensors capable of quantifying both metals in a single readout remains challenging. Second, portable devices require independence from centralized laboratories, expensive instrumentation, and highly trained personnel.^11^^,^^12^^,^^13^ This necessitates a user-friendly low-volume operation platform integrating a compact, temperature-controlled incubation module to preserve biosensor viability, alongside a transition module that directly converts visual readouts into numerical values without professional software or instrumentation, which remains a key challenge in biosensor design.

CadR acts as a Cd-binding transcriptional activator that induces fluorescent reporter expression in whole-cell biosensors for metal quantification. Its architecture resembles that of MerR, a regulator involved in Hg detoxification,^14^^,^^15^ and CadR, therefore, also responds to Hg. However, divergent resistance mechanisms and metal-binding affinities among bacterial species generate heterogeneous dose-response curves for Cd^2+^ and Hg^2+^,^14^^,^^16^^,^^17^ complicating their simultaneous detection. Thus, selecting and engineering a chassis organism capable of collective detection of total highly toxic metals remains a critical unmet goal. As reported,^11^ the collective quantification here means quantifying the total concentration of two highly toxic metals, Cd and Hg, in a single sample droplet within one assay. Additionally, as highlighted in recent studies,^11^^,^^18^^,^^19^^,^^20^^,^^21^ translating fluorescent signals into a visual output readable by the naked eye or consumer electronics is equally essential. While numerous cell-free sensor platforms^11^^,^^18^^,^^22^^,^^23^^,^^24^^,^^25^^,^^26^ and several whole-cell biosensor devices for heavy metals^5^^,^^6^^,^^7^^,^^27^^,^^28^^,^^29^^,^^30^^,^^31^^,^^32^^,^^33^^,^^34^ have been reported, none of them simultaneously fulfill the following requirements: collective quantification of highly toxic Cd^2+^ and Hg^2+^, user-friendly operation for non-specialists, integrated temperature-controlled incubation for field-based bacterial viability, and seamless conversion of visual readouts to numerical values without external data processing. Thus, there remains an urgent need for an integrated, portable device that meets the above requirements.

**Figure 1:**
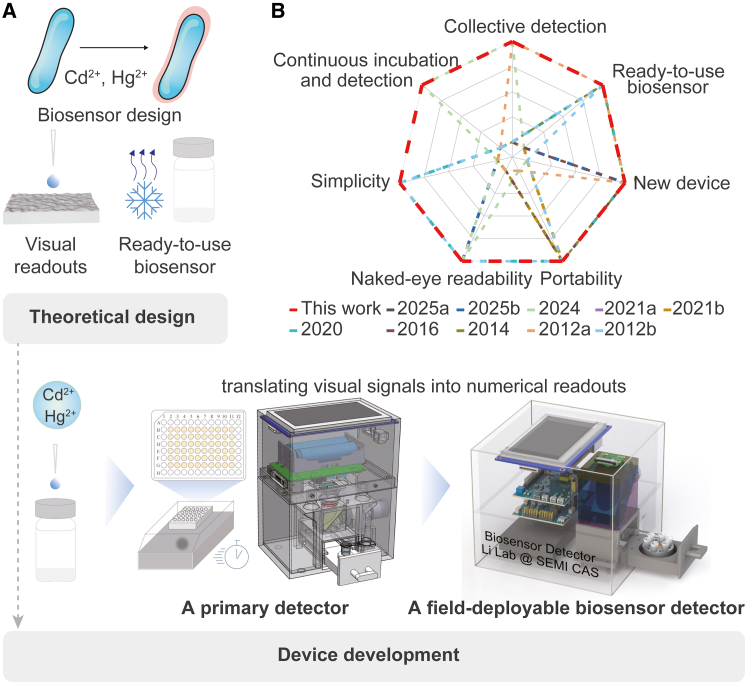
Development of a field-deployable whole-cell biosensor for the on-site quantification of highly toxic heavy metals (A) Workflow from theoretical design to functional device. (B) Radar plot compares key performance metrics of our biosensor device against other published devices.

In this work, we merge the best attributes of a whole-cell biosensor with naked-eye readability to create a field-deployable platform for simultaneous Cd^2+^ and Hg^2+^ detection (Figure 1A). Compared with existing whole-cell sensor devices for heavy metal detection, the integrated platform reported here uniquely enables genuine field deployment (Figures 1B; Table S1). Firstly, we engineered *Vibrio natriegens* into a whole-cell biosensor by introducing a compact gene circuit comprising a CadR-based sensing module and an mCherry-based reporter module. *V*. *natriegens* was chosen^35^^,^^36^^,^^37^ (1) for its exceptional tolerance to toxic contaminants, allowing simultaneous detection of highly toxic metals, and (2) for its adaptability to complex environments, a prerequisite for on-site, whole-cell biosensing. Secondly, we lyophilized this biosensor into a stable powder that retains activity for months and reactivates within minutes upon water sample addition. This design eliminates field-based live-cell maintenance and complex pre-incubation procedures. Thirdly, a microdroplet concentrator was developed to amplify the biosensor’s visual signal. Finally, the complete system is packaged into a palm-sized, disposable cartridge requiring no specialized operation and stable under ambient conditions, and achieves the transition of visual readouts from images to numerical values. Therefore, this portable device simplifies the operation, storage, and distribution of biosensors, enabling the direct visualization of highly toxic contaminants. It particularly benefits non-specialist users in low-resource areas and enables collective quantification of Cd^2+^ and Hg^2+^ in real river water samples. We also calculate the cost of this biosensor device for fabrication and single detection. By bridging biosensor design and device development, this work establishes a direct pathway from genetic circuit to handheld deployment for the *in-situ* monitoring of co-occurring toxic metals in contaminated water.

## Results

### Biosensor blueprint establishment

**Figure 2:**
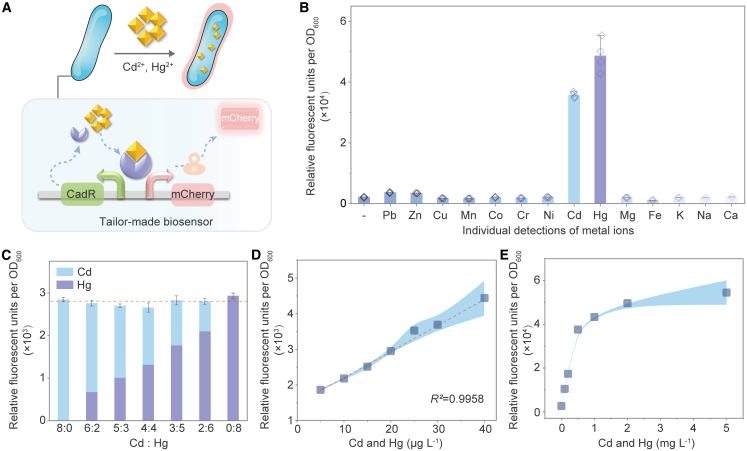
Collective detection of highly toxic metals by our whole-cell biosensor (A) Mechanism of the biosensor detection for highly toxic metals. (B) Fluorescent output upon exposure to 20 μM of indicated metal ions. Cd^2+^: ∼2.2 mg L^−1^; Hg^2+^: ∼4 mg L^−1^. Data are presented as mean ± s.d. (*n* = 4, biologically independent samples). (C) Individual detection of Cd^2+^and Hg^2+^, and collective detection of two metal ions at varying Cd^2+^:Hg^2+^ mixing ratios, each at a total concentration of 20 μg L^−1^. Column color indicates mixing ratio. (D and E) Data are presented as mean ± s.d. (*n* ≥ 3). Dose-response curves for the biosensor exposed to a Cd^2+^:Hg^2+^ (1:1) mixture at total concentrations in the (D) μg L^−1^ and (E) mg L^−1^ ranges. Data are presented as mean ± s.d. (*n* = 4).

To enable collective detection of highly toxic metals, we engineered *V. natriegens* with a compact genetic circuit containing a CadR-based sensing module and an mCherry-based reporter module (Figure 2A). This tailored biosensor produces red-fluorescent outputs scaled to metal concentrations upon CadR-toxin binding. It exhibits ≈10-fold higher fluorescence response to Cd^2+^/Hg^2+^ versus other metals at equivalent concentrations (Figure 2B). Remarkably, the signal outputs for the individual and collective detection of Cd^2+^/Hg^2+^ at identical total concentrations show 2.30%–5.29% deviation (Figures 2C and S1A), enabling collective quantification of these two metals in a single assay (*R*^*2*^ = 0.9958, Figure 2D). This capacity, likely stemming from the distinctive induction efficiency of the regulatory factor in our *V. natriegens*-based biosensor, contrasts with other bacterial chassis that carry different detoxification pathways and metal-binding affinities.^14^^,^^15^ The biosensor maintains robust growth (<5% deviation) across working metal concentrations (0−5 mg L^−1^) (Figures S1B–S1C). The biosensor can collectively quantify highly toxic metals within each independent batch (Figures 2E and S1D); the observed differences between batches arise from inherent biological variability. Collectively, these results establish a platform for the simultaneous detection of highly toxic metals in complex aqueous samples by the whole-cell biosensor.

### Developing a hydrophobic concentrator for biosensor signal amplification

**Figure 3:**
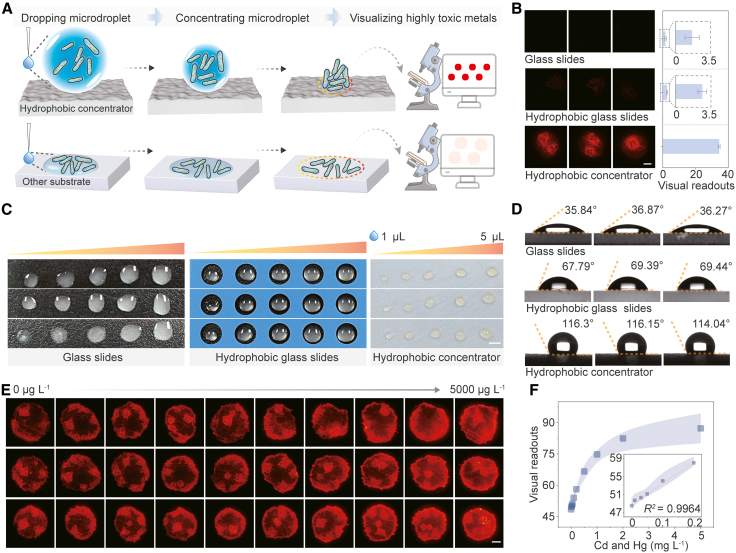
Signal amplification through enhanced microdroplet confinement on a customized hydrophobic concentrator (A) Schematic illustration of biosensor microdroplets aggregation on different substrates. Orange dashed lines demarcate the contact areas. (B) Fluorescence micrographs (left) and corresponding visual readouts (right) of identical analyte volumes (3 μL) applied to different substrates. Imaging parameters and exposure time were fixed across replicates, and images were processed with ImageJ. Scale bars, 500 μm. Data are presented as mean ± s.d. (*n* = 3). (C) Optical images of microdroplets (1−5 μL) on different substrates, illustrating initial contact areas. Scale bars, 0.25 cm (D) Quantitative assessment of substrate hydrophobicity via static contact angle measurements. (E) Fluorescence micrographs of microdroplets (3 μL) containing graded concentrations of mixed toxic metals (Cd^2+^:Hg^2+^ = 1:1, 0−5000 μg L^−1^) on the hydrophobic concentrator (fixed imaging parameters). Scale bars, 500 μm (F) Visual detection range of mixed toxic metals (Cd^2+^:Hg^2+^ = 1:1), quantified from (E) using ImageJ-processed readouts. Data are presented as mean ± s.d. (*n* = 3).

Visual detection provides a user-friendly and early-warning system for pollution, enabling non-professionals to detect toxic metals via naked eye observation or smartphone imaging.^22^ We developed a hydrophobic concentrator that amplifies whole-cell biosensor fluorescence by concentrating analytes at the substrate interface through microdroplet volume reduction (Figures 3A and S2). This concentrator enhances signal intensity 21-fold versus commercial glass slides and 13-fold versus commercial hydrophobic glass slides (Figure 3B). While commercial hydrophobic glass slides poorly confine microdroplets (Figure 3C), our concentrator’s superior hydrophobic confinement shrinks droplets to focused focal points (Figure 3D), ensuring full-field fluorescence capture for visual detection. The concentrator exhibits excellent uniformity and batch reproducibility (deviation of <5%) (Figure S3) and can be recycled/deactivated post-use for safe disposal. Quantitative imaging shows a linear correlation (*R*^*2*^ = 0.9964) between heavy metal concentration (0−200 μg L^−1^ range) and fluorescence intensity (Figures 3E and 3F) with high reproducibility (Figure S4), establishing a simple platform for visual toxic metal detection.

### Lyophilized biosensor for ready-to-use simplicity

**Figure 4:**
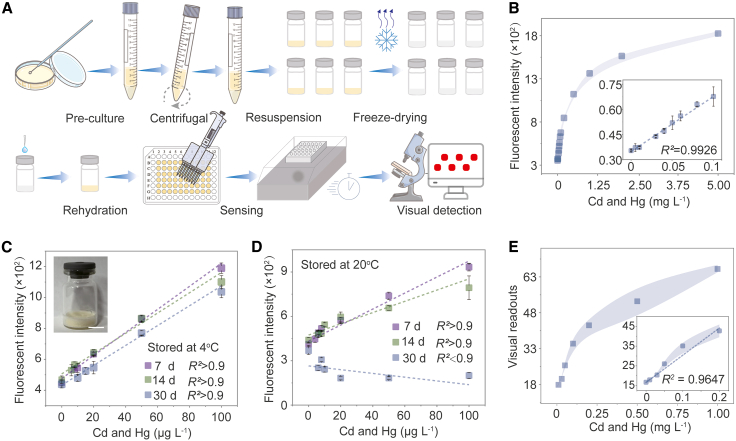
Ready-to-use simplicity via lyophilized biosensor in portable vials with water reactivation (A) Preparation and use workflow for the lyophilized biosensor. (B) Dose-response of lyophilized biosensor to mixed toxic metals (Cd^2+^:Hg^2+^ = 1:1). Data are presented as mean ± s.d. (*n* = 4). (C and D) Stability assessment of lyophilized biosensor after 7, 14, and 30 days of storage at 4°C and 20°C. Data are presented as mean ± s.d. (*n* = 4). Inset shows a vial containing the lyophilized biosensor. Scale bars, 1 cm (E) Visual detection range of mixed toxic metals (Cd^2+^:Hg^2+^ = 1:1), quantified from images (See also Figure S5C) using ImageJ-processed readouts. Data are presented as mean ± s.d. (*n* = 3).

Conventional whole-cell biosensors require laborious, expert-driven pre-incubation steps^38^, rendering them impractical for non-specialists. To address this limitation, we converted our designed sensor into a pre-prepared, freeze-dried, and storable reagent, a portable biosensor housed in a compact bottle (diameter 2.2 cm, height 3.5 cm, Figure 4A). Our results demonstrate that this portable biosensor can collectively detect two highly toxic metals (Figure 4B). Simple rehydration with water samples eliminates all cultivation steps, removing the need for specialized equipment and trained personnel while saving time. The lyophilized formulation exhibits a satisfying storability, retaining quantitative ability for highly toxic metals after 30 days of storage at 4 °C and about 14 days at 20 °C (Figures 4C and 4D), but deteriorates within one week at 30 °C (Figure S5A). Signal amplification was increased ∼10-fold by resuspending the collected biosensor in a one-half dilution of the residual supernatant, followed by freeze-drying (Figure S5B). We further demonstrated that the biosensor enables the visual quantification of highly toxic metals (Figures 4E and S5C), maintaining reliability in contaminant-spiked river water (Figure S6). With its simplicity, portability, and storability, this biosensor provides timely alerts for non-professionals in resource-limited settings.

### Development of an integrated field-deployable biosensor detector

Reliance on fluorescence microscopes and bench-top oscillators compromises biosensor portability for visual detection. While handheld illuminators paired with smartphone applications^11^^,^^21^^,^^22^ and portable fluorescence spectrometers^39^ address partial needs, our two-stage solution integrates multiple devices and eliminates both bottlenecks.

**Figure 5:**
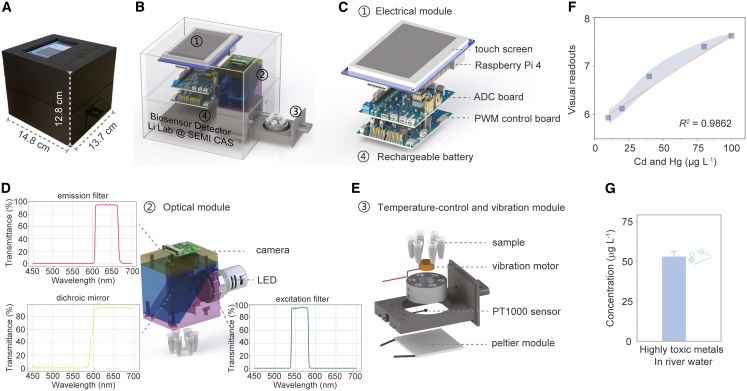
Integrated field-deployable biosensor detector for highly toxic metal detection (A) Assembled handheld device. (B–D) (B) Exploded view of four modules: (C) electrical module with rechargeable battery, (D) optical module, and (E) temperature-control and vibration module. (F) Calibration curve (See also Figure S8A). Data are presented as mean ± s.d. (*n* = 4). (G) Validation of the detector using river water samples spiked with 50 μg L^−1^ highly toxic metals. Data are presented as mean ± s.d. (*n* = 4).

***Stage 1* - *Hand-held prototype.*** We fabricated a palm-sized detector (11.8 cm × 8.3 cm × 15.3 cm) to replace a fluorescence microscope, which is a heavy and cumbersome instrument and also requires computer-assisted image processing for translating visual readouts from image to values (Figure S7A). This allows us to quantify highly toxic metals after mixing real water samples using a commercial mini-oscillator. Crucially, it demonstrated inherent resistance to Zn^2+^ interference, a common interferent in Cd^2+^ and Hg^2+^ monitoring,^17^^,^^40^ delivering quantitative results indistinguishable from bench-top assays (*P* > 0.05, Figures S7B–S7D) when tested with Zn^2+^-containing water samples. Similar findings were observed for Pb^2+^ interference tests (*P* > 0.05).
***Stage 2* - *All-in-one field detector.*** The upgraded device (13.7 cm × 14.8 cm × 12.8 cm) integrates a six-well, temperature-controlled shaker, eliminating the need for an external oscillator (Figure 5A). Figures 5B–5E detail the upgraded detector’s architecture: a single, spatially optimized chassis seamlessly combines sensing, control, and actuation modules, creating a compact device ready for portable, field deployment. This upgraded device exhibits the detection limit of 10 μg L^−1^ for highly toxic metals and a linear quantification range of 10–100 μg L^−1^ (*R*^*2*^ = 0.9862) at a mixing ratio of 1:1 (Figures 5F and S8A–S8C), providing direct numerical readouts of contamination levels. The recovery rate (detected concentration/known concentration) was 97.74%–105.84% at the test concentration in real river water samples (Figures 5G and S8D).

In summary, these handheld and all-in-one configurations provide non-specialists with a simple and portable tool for rapid on-site assessment of highly toxic metals in natural waters. Our work establishes a practical pathway for translating theoretical biosensor designs into field-deployable detectors.

## Discussion

We describe a complete pipeline, from synthetic biology design to a field-deployable biosensor detector, enabling non-professionals to visually quantify highly toxic metals in polluted waters simply and conveniently. Our biosensor chassis tolerates high salinity,^41^ growing normally in 22‰ NaCl medium,^42^ while retaining full detection of toxic analytes, thereby removing a key barrier^22^ to further deploying whole-cell sensors in commonly salt-rich contaminated waters. The biosensor can tolerate total toxic metal concentrations up to 5 mg L^−1^ and enable collective quantification of Cd^2+^ and Hg^2+^ at concentrations as low as 10−100 μg L^−1^ in real river water samples. Samples beyond this range require dilution before measurement. When paired with the palm-sized detector, the assay eliminates the need for laboratory infrastructure or trained personnel. Operation simply involves adding the contaminated sample, and a numeric readout is then obtained by non-specialists. The system supports two calibration modes. For rapid on-site pollution assessment, a set of lyophilized standards allows users to match the displayed value to a discrete contamination range. For higher precision, a separate set of standards generates an internal calibration curve, which converts the signal into absolute concentration to ensure quantitative results in the field.

The estimated cost of our developed biosensor device is approximately $401.5, which is significantly lower than that of non-portable laboratory instruments (e.g., ICP-MS, microplate reader, or fluorescence microscope), typically priced above $10,000. These conventional instruments are used for the direct detection of highly toxic metals or indirect detection of biosensor fluorescence. Our device is also more cost-effective than portable metalyser and luminometers reported in previous studies^6^^,^^43^ for on-site metal detection. The cost of detection consumables for our device is approximately $0.33 per test, including disposable biosensors (∼$0.06), 200-μL tubes (∼$0.05), and transfer pipettes (∼$0.22) required for one batch of six-channel detection. This per-test cost is slightly lower than that of commercially available field detection devices (ranging from $0.5 to $11.3).^6^^,^^43^ Our biosensor device’s cost advantage is attributed to the device’s compact size, minimal sample volume requirements, and simple operational procedures. The platform is readily adaptable to other emerging pollutants or complex toxic contaminants. Collectively, this work delivers an early-warning system that translates biosensor theory into a practical, *in situ* tool for resource-limited communities and establishes a generalizable framework for portable toxicant detection.

### Limitations of the study

Current limitations in response time, detection limit (Table S5), and signal intensity of background and sample are being addressed by accelerating analyte uptake via periplasmic retargeting of sensing elements, faster gene circuits, and optimized preparation and detection protocols. Biosensor performance can be improved by several cutting-edge, circuit-level synthetic-biology strategies, including genetic logic circuits,^44^ cascaded amplifying circuits,^45^ *trans*-splicing denoiser circuits,^46^ and modular, programmable sensor architectures^47^ to better meet practical detection demands. Background signals can be reduced by minimizing the basal expression of the genetic circuits in the sensor cells; detector signal intensity can subsequently be enhanced by component optimization, such as using a stronger light source to accommodate more channels.

## Resource availability

### Lead contact

Requests for further information and resources should be directed to and will be fulfilled by the lead contact, Lu Lu (lulu@hit.edu.cn).

### Materials availability

Materials generated in this research will be made available on request, but we may require a payment and/or a completed materials transfer agreement if there is potential for commercial application.

### Data and code availability

• Data in this research will be made available on request.
• This article does not report original code.
• Any additional information required to reanalyze the data reported in this article is available from the lead contact upon request.

## Acknowledgments

This work was supported by the 10.13039/501100012166National Key Research and Development Program of China (2024YFB4105700), the 10.13039/501100001809National Natural Science Foundation of China (52470071, 52321005, 32230060, 52200090, and 52200048), and the Shenzhen Science and Technology Program (JCYJ20220818101804010, RCYX20221008092901004, and ZDSYS20220606100606013).

## Author contributions

Conceptualization, L.L., X.G., and Z.L.; methodology, S.P., L.F., Q.W., W.Y., and S.Y.; investigation, S.P., L.F., Q.W., W.Y., and S.Y.; writing – original draft, S.P., L.F., and Q.W.; writing – review and editing, L.L., X.G., and Z.L.; funding acquisition, L.L., X.G., Z.L., S.P., and L.F.; resources, L.L., X.G., and Z.L.; supervision, L.L., X.G., and Z.L.

## Declaration of interests

L.L., X.G., Z.L., S.P., Q.W., W.Y., and S.Y. are co-inventors on filed China patents CN114807291B and CN202410695751.3 related to the design of the whole-cell biosensor and the detection device that incorporates discoveries included in this manuscript. The remaining authors declare no competing interests.

## STAR★Methods

### Key resources table

**Table:** 

REAGENT or RESOURCE	SOURCE	IDENTIFIER
**Bacterial and virus strains**		

*V. natriegens* VnDx, see Table S2	Xu et al.,^12^	N/A
*V. natriegens* XG210, see Table S2	This work	N/A

**Recombinant DNA**		

pET-21a, see Table S2	Novagen	Cat#69740
pPcad-mcherry, see Table S2	Guo et al.,^13^	N/A

**Software and algorithms**		

ImageJ	Schneider et al.,^48^	https://imagej.net/ij/(https://imagej.nih.gov/ij/)

**Other**		

Microplate reader (Synergy H1)	Agilent BioTek	https://www.agilent.com.cn/zh-cn/product/microplate-instrumentation/microplate-readers
Fluorescence microscope DP47	Olympus	https://evidentscientific.com/en/life-science-microscopes

### Experimental model and study participant details

#### Strains of microorganisms

*V. natriegens* XG210, served as the whole-cell biosensor in this work, was obtained for sensing highly toxic metals via engineering *V. natriegens* VnDx. All experiments were conducted using strains stored in glycerol at −80 °C.

### Method details

#### Whole-cell biosensor construction

The whole-cell biosensor was constructed by genetically engineering *Vibrio natriegens* following the procedure reported in ref.^36^ Specifically, the gene coding the transcriptional regulator CadR, along with the divergent *cad* promoter,^15^ was chemically synthesized by GenScript. Subsequently, this gene construct was cloned together with a promoterless *mCherry gene* into the plasmid vector pET-21a, yielding the plasmid pPcad-mcherry. The plasmid pPcad-mCherry, designed for sensing cadmium and mercury, was then introduced into *V. natriegens* VnDx, resulting in the engineered strain designated as *V. natriegens* XG210, which served as the whole-cell biosensor in this work. Detailed information on bacterial strains and plasmids is provided in Table S2. LBv2 medium (per 1 L) contains LB powder 25 g, NaCl 11.9 g, KCl 0.313 g, and MgCl_2_ 2.2 g, and medium plates were prepared by adding 1.5% agar.

#### Potential metal interference in collective detection

Strain XG210, stored in glycerol at −80°C, was revived by streaking on LBv2 agar plates. A single colony was then inoculated into LBv2 liquid medium and cultured at 37°C, and then cells were harvested for heavy metal detection. To analyze potential metal interference, the biosensor was exposed to a panel of metal ions commonly found in polluted water, including Na^+^, K^+^, Ca^2+^, Mg^2+^, Fe^3+^, Pb^2+^, Zn^2+^, Cu^2+^, Mn^2+^, Co^2+^, Ni^2+^, and Cr^3+^, each at a concentration of 20 μM. The fluorescence intensity, serving as the signal output, was quantified by calculating RFU, the relative fluorescent intensity per unit OD_600_^16^. Fluorescence intensity and OD_600_ were measured using a microplate reader (Synergy H1, Agilent BioTek) at 37°C. The fluorescence intensity was recorded at an excitation wavelength of 580 nm and an emission wavelength of 610 nm, indicating the expression level of red fluorescent protein (mCherry) induced by the presence of metal ions. The OD_600_ was measured to determine the cell density.

#### Collective quantification of highly toxic metals

To collectively quantify highly toxic metals, we performed individual detection of Cd^2+^ (20 μg L^−1^) and Hg^2+^ (20 μg L^−1^), as well as collective detection of Cd^2+^ and Hg^2+^ (the total concentration of 20 μg L^−1^) in a single test to assess the consistency of detection. Specifically, the collective detection of Cd^2+^ and Hg^2+^ was prepared by mixing them at ratios of 6:2, 5:3, 1:1, 3:5, and 2:6, respectively, while maintaining the same total concentration. Response time of the biosensror was ∼11.5 h, which is the elapsed time from the moment the analyte contacts the sensor until the output signal reaches 90% of its final steady-state value. The fluorescence signal was routinely read at 12 h, and the detection temperature is 37°C. To determine the range of collective quantification by the biosensor, it is exposed to different total concentrations of Cd^2+^ and Hg^2+^ at a mixing ratio of 1:1, under the same detection conditions as mentioned above.

#### Preparation of hydrophobic concentrator

A hydrophobic concentrator was prepared as previously reported,^11^ by cutting a commercially available PTFE film to the required size, followed by rinsing, roughening, rinsing again, and drying with a gentle stream of nitrogen gas. To evaluate its superiority in visual detection, the concentrator’s performance in microdroplet aggregation, hydrophobicity, and signal output intensity was compared with other commercially available substrates (glass slides and hydrophobic glass slides). The capability to aggregate microdroplets was assessed by capturing images after dispensing respective 1−5 μL microdroplets of the same analytes onto each substrate. Hydrophobicity was evaluated by measuring the static contact angles on each substrate using an optical contact angle goniometers (OCA 20, Dataphysics). Signal output performance was assessed by comparing the visual readout values of the same analytes on different substrates. Images were captured under consistent exposure conditions after dispensing respective 1−3 μL microdroplets of the same analytes onto each substrate. To assess the uniformity of the prepared hydrophobic concentrators, hydrophobicity was measured at a minimum of eighteen random sites on three separate batches of hydrophobic concentrators. Statistical analysis was performed to evaluate the deviation in hydrophobicity across these sites.

#### Visual detection using fluorescence microscope

To visually detect highly toxic metals, a series of analyte microdroplets (3 μL each) were dropped onto a hydrophobic concentrator. After drying, images were captured at a fixed exposure time using a fluorescence microscope (DP47, Olympus). The visual readouts, represented as the Intensity/area value, were subsequently analyzed using ImageJ software.^48^ Here, intensity/area denotes the relative fluorescent intensity per unit area within the images. To process the images, we first cropped them to a fixed area and then split the color channels. The intensity and area of the analyte images in the red channel were measured, which indicated the expression of the red fluorescent protein (mCherry) in the biosensor in response to the highly toxic metals. Calibration curves were constructed by correlating the visual readouts with the known concentrations of the analytes.

#### Preparation and evaluation of the portable biosensor

To prepare the portable biosensor, our developed whole-cell biosensors were freeze-dried following a previously reported method.^6^ Specifically, the biosensors were cultured, collected and resuspended in 150 mL of cryoprotectant solution (LBv2 medium supplemented with 10% trehalose and 1.5% polyvinylpyrrolidone). Each 1 mL aliquot was transferred into a 5 mL vial, cooled at −80 °C for 1 h, and then lyophilized for 16 h. To demonstrate the performance of the portable biosensor in detecting highly toxic metals, the freeze-dried biosensors were rehydrated with water samples and analyzed using a microplate reader as described above. To assess the long-term storability of the portable biosensor, hundreds of vials were stored at 4 °C, 20 °C, and 30 °C, respectively. The performance of the biosensors in quantifying highly toxic metals was evaluated periodically over a period of at least 30 days. To optimize the signal output, the lyophilization components were adjusted by retaining one-half of the residual supernatant from the collected biosensor cultures. The responses of the biosensors to highly toxic metals were measured as signal output using a microplate reader. The portable biosensor prepared with the optimized lyophilization components was then rehydrated was used for visual detection of highly toxic metals, as described above. To evaluate the feasibility of visual detection in river water, the portable biosensor was rehydrated with river water spiked with highly toxic metals (0−1000 μg L^−1^). Real river water was collected from Dasha River (Shenzhen, China), filtered to remove sediments, and used to rehydrate the portable biosensor. A commercially available portable oscillator (DLAB Scientific Co., Ltd.) was used to mix the rehydrated biosensor with river water spiking with highly toxic metals. No cadmium or mercury was detected in the raw river water by ICP-MS. Visual readouts were recorded using a fluorescence microscope after depositing microdroplets onto a hydrophobic concentrator, as described above.

#### Fabrication and testing of the primary detector

The design of the primary detector for biosensor detection is detailed in Figure S9A. This detector integrates advanced hardware with sophisticated software to measure fluorescence intensity. Specific components used in the fabrication of the detector are listed in Table S3. The casing, frame, and chip slot were custom-made using a laboratory 3D printer (Bambu lab X1) with final dimensions of 11.8 cm × 8.3 cm × 15.3 cm. The internal detection principle is illustrated in Figure S9B. As shown in Figure S9C, the core of the detector includes a driverless LED module (Ruibao Tech, Shenzhen, China) that emits green-yellow light (560–565 nm) to excite the mCherry protein. After detection, the fluorescence signal from the mCherry protein in a 200-μL reaction tube is captured by a high-resolution camera (Waveshare electronics) at a user-defined intensity threshold. The average fluorescence intensity is then calculated from the recorded image and displayed on the screen. This process is facilitated by customized software running on a Raspberry Pi 4 (Waveshare electronics), which processes the image data in the HSV color space. The portable biosensor was rehydrated with the aforementioned river water samples spiked with highly toxic metals and mixed using a commercially available portable oscillator. Detection was performed using the primary detector. To assess potential metal interference, either additional Zn^2+^ (200 μg L^−1^) or Pb^2+^ (20 μg L^−1^) was spiked into real river water samples containing highly toxic metals (20 μg L^−1^), respectively.

#### Development of the field-deployable biosensor detector

The field-deployable biosensor detector was fabricated to detect contaminated water. It integrates an *in-situ* vibration module, a temperature-controlled module, and a six-sample incubator to upgrade the primary detector described above, thereby eliminating the need for an additional oscillator in visual detection. After rehydration with the aforementioned real river water spiked with highly toxic metals, the portable biosensor was incubated at 37 °C. Images were captured at 8 h, and the response time was determined to be 7.5 h. Detailed information on the fabrication of the field-deployable biosensor detector, including part names, suppliers, part numbers, unit costs, and technical specifications, is provided in Table S4. Mechanical components were either 3D printed in-house using a Bambu Lab X1 printer or machined via CNC by JLCNC. All electronic modules, including a custom PCB for PWM signal routing, were manually assembled. The modular design enables easy replication and supports future system upgrades and expansions in laboratory settings. The specific modular information can be obtained as following:

##### System architecture

The field-deployable biosensor detector features a dual-stack modular architecture housed within a compact, 3D-printed enclosure. On the rear side, the electrical module consists of a 4.3-inch capacitive touchscreen and a Raspberry Pi-based control unit, which are vertically aligned above a rechargeable lithium battery (UPS HAT module) to enable autonomous operation. On the front side, the optical module is coaxially aligned with the temperature-control and vibration module, establishing a direct excitation-to-detection optical pathway for fluorescence imaging. The temperature-control and vibration module holds six 200-μL reaction tubes arranged in a circular pattern, integrating temperature regulation and mechanical vibration. All structural components were fabricated in-house using fused deposition modeling (Bambu Lab X1), while the optical and mechanical alignment frames were precision-machined from aluminum.

##### Electrical module

The electrical module consists of four vertically stacked layers within a compact footprint. At the top, a 4.3-inch capacitive touchscreen serves as the user interface for experimental setup and real-time monitoring. Directly below, the Raspberry Pi 4 board functions as the central controller, executing Python-based programs for image acquisition, PWM signal generation, and thermal regulation. Analog input signals, such as those from a PT1000 RTD sensor, are digitized using a high-precision AD HAT to ensure accurate temperature measurement. At the base, a custom-developed PWM control circuit board distributes drive signals to the LED, Peltier element, and vibration motor. This PWM control board was fabricated by JLCPCB and assembled manually.

##### Optical module

The optical module integrates fluorescence excitation, spectral filtering, and image acquisition into a coaxially aligned structure. Side-incident excitation light from a 560–565 nm LED first passes through a 560 ± 20 nm narrow-bandpass filter and is then reflected by a 590 nm long-pass dichroic mirror onto the sample. Fluorescence emitted from the sample travels upward through a 635 ± 20 nm emission filter before reaching a top-mounted CMOS camera. All optical components are precisely mounted in a CNC-machined aluminum holder to ensure precise optical alignment. This module is vertically aligned with the temperature-control and vibration module below, enabling real-time fluorescence detection during thermal cycling or isothermal reactions.

##### Temperature-control and vibration module

The temperature-control and vibration module integrates incubation, temperature regulation, and mechanical agitation into a vertically aligned structure. Six 200-μL reaction tubes are housed in a cylindrical aluminum incubator designed to ensure uniform thermal contact. A miniature eccentric vibration motor is centrally mounted to provide consistent and reliable mixing. Thermal feedback is provided by a platinum RTD (PT1000 sensor), which is inserted laterally into the thermal core through a precision-machined bore. Heating and cooling are facilitated by a thermoelectric Peltier element located beneath the aluminum incubator. All components, operated under PWM control, are regulated by feedback algorithms executed on the Raspberry Pi.

### Quantification and statistical analysis

All data were analyzed from at least three independent experiments. Data were expressed as the mean ± standard deviation (SD). *p* value was determined by a two-tailed unpaired *t* test with difference significance at ∗*p* < 0.05, ∗∗*p* < 0.01, ∗∗∗*p* < 0.001, and the statistical method is detailed in the corresponding figure legends.
