# Supplementary material for: Visual detection of collective highly toxic metals in water by a handheld whole-cell biosensing detector: From circuit to field
Source: iScience. 2026 Mar 10;29(4):115289. doi: 10.1016/j.isci.2026.115289 (PMC13049442; doi:10.1016/j.isci.2026.115289)
Supplement: Document S1. Figures S1–S9 and Tables S1–S5 [file mmc1.pdf]

iScience, Volume *29*

Supplemental information

Visual detection of collective highly toxic metals in water by a handheld whole-cell biosensing detector: From circuit to field

Shanshan Pi, Liang Feng, Qiongzhang Wang, Wenjun Yang, Shanshan Yu, Zhao Li, Xiang Gao, and Lu Lu

Supporting Information

Supplementary Table 1 | Comparison of the reported whole-cell biosensor devices for detecting heavy metals.

Devices	Detection of Cd and Hg	Ready-to-use sensor	New device	Portability of device	Naked-eye readability	Simplicity for non-specialists use	Continuous detection, temperature-controlled incubation
This work	Y	Y	Y	Y	Y (numbers)	Y	Y
2025a[1]	N (Pb)	N (pre-culture)	Y (PlomBOX)	Y	N (images)	N (cell revival)	N
2025b[2]	N (As)	N (pre-culture)	Y (CMOS chip)[3]	Y	Y (numbers)	N (cell revival)	N
2024[4]	Y (CdPbHg)	N (pre-culture)	N (lab microplate reader)	N	Y (numbers)	N (cell revival and lab instruments)	Y
2021a[5]	N (Hg)	Y	Y (orthogonal paper)	Y	N (images)	N (image process)	N
2021b[6]	N (Cd)	N (pre-culture)	N (lab LED plate)	Y	N (images)	N (cell revival and image process)	N
2020[7]	N (Hg)	Y	Y (test strips)	Y	Y (color comparison)	Y	N
2016[8]	N (As)	N (pre-culture)	Y (microfluidic biochips)	Y	N (images)	N (cell revival and image process)	N
2014[9]	N (As)	Y	Y (integrated microfluidic cell chip)	Y	N (electronic signals)	N (signal process)	N
2012a[10]	Y (CdHgCuZn)	Y	Y (Lumisens III and IV)	N	N (images)	N (image process)	N
2012b[11]	N (As)	Y	N (lab luminometer)	Y	Y (numbers)	Y	N

Supplementary Table 2 | Detailed strains and plasmids used in this work.

Strains and plasmids	Description	Source
**Bacterial strains**		
Vibrio natriegens VnDx	Derived from ATCC 14048 by integrating the T7 RNA polymerase expression cassette at the dns locus	[12]
V. natriegens XG210	VnDx harboring pPcad-mcherry	This work
**Plasmids**		
pET-21a	Amp^R, T7 promoter, lac operator	Novagen
pPcad-mcherry	pET-21a derivative containing *cadR*, Pcad divergent promoter region, and promoterless mcherry	[13]

Supplementary Table 3 | Details of parts used in fabricating the primary detector.

Parts	Suppliers	Cost ($)	Details
Driverless LED module with a heat sink	Ruibao Tech, Shenzhen, China	1.43	green-yellow light (560-565 nm), 3-5v, 3w, 5°
Narrow band-pass filter (560 nm ± 5 nm)	Rayan Tech, Changchun, China	38.92	No. BP560-10-D25.4×6; D25.4×6mm-OD5-T90%
Narrow band-pass filter (635 nm ± 5 nm)	Rayan Tech, Changchun, China	38.92	No. BP635-10-D25.4×6; D25.4×6mm-OD5-T90%
Dichroic filter (590nm)	Rayan Tech, Changchun, China	38.92	No. FLU-LP590; 25.2 × 35.6 × 1mm
Raspberry Pi 4	Waveshare electronics	54.1	No. 16784; PI4B-2GB
Capacitive Touch Display for Raspberry Pi	Waveshare electronics	35.99	No. 16239; 4.3 inch, DSI Interface, 800×480
Uninterruptible Power Supply UPS HAT (B) for Raspberry Pi	Waveshare electronics	22.99	No. 20567; 5V Output, up to 5A Current, Pogo Pins Connector
High-resolution camera for Raspberry Pi	Waveshare electronics	29.99	No. 22652; IMX519-78 16MP AF Camera, Auto-Focus, 78.5° FOV
3D structure	Laboratory 3d printer(Bambu lab X1)	4.96	Top case, $ 1.65<br>Midframe, $ 1.42<br>Chip slot, $ 0.26<br>Bottom case, $ 1.63

Supplementary Table 4 | Details of parts used in fabricating the field-deployable biosensor detector.

Parts	Supplier, part number	Cost ($)	Technical Details	Remarks
3D printed housing	In-house (Bambu Lab X1)	~2	Top case, sample loading slot, bottom case; FDM; black PLA	Printed and assembled in-house
Custom PCB board	JLCPCB (Shenzhen, China)	~3	2-layer PCB for PWM and ADC signal routing	Controls LED, Peltier, and motor
Raspberry Pi 4	Waveshare Electronics, 16784	53	Pi 4B, 2 GB RAM, Linux-based system	Central control unit
High-Precision AD HAT	Waveshare Electronics, 18983	25	32-bit ADC (ADS1263), 10 channels, SPI interface	For PT1000 RTD signal acquisition
Capacitive touch display	Waveshare Electronics, 16239	31	4.3inch, 800×480 pixels, DSI interface	Main user interface
UPS HAT module	Waveshare Electronics, 20567	20	5 V output, max 5 A, pogo pin connector	Power backup for mobile operation
High-resolution camera	Waveshare Electronics, 22652	41	16 MP, IMX519, autofocus, 78.5° field of view	Top-down fluorescence imaging
LED excitation module	Ruibao Tech, (Shenzhen, China)	1.5	Green-yellow light (560–565 nm), 3W, 5° beam angle	With built-in heat sink
Excitation filter (560 nm)	Rayan Tech, (Changchun, China), BP560-10-D25.4x6	40	Center 560 nm ±20 nm, Ø 25.4×6 mm, T > 90%	Narrow-bandpass filter
Emission filter (635 nm)	Rayan Tech, (Changchun, China), BP635-10-D25.4x6	40	Center 635 nm ±20 nm, Ø 25.4×6 mm, T > 90%	Narrow bandpass filter
Dichroic mirror (590 nm)	Rayan Tech, (Changchun, China), FLU-LP590	40	Cutoff 590 nm, 25.2×35.6×1 mm	Separates excitation and emission paths
Optical module casing	JLCNC (Shenzhen, China)	~100	CNC-machined aluminum frame	Optical alignment housing
Peltier module	Xinwei Electronics (Jiaxing, China)	3	Max 60 W, bidirectional heating/cooling	Mounted beneath sample chamber
RTD (PT1000) sensor	Jukong Tech (Zhengzhou, China), P-M-A-1/4-6-T-3	1	Class A platinum RTD, –200 °C to 200 °C, SUS304	Laterally inserted into thermal center
Vibration motor	Kebei Electronics (Shenzhen, China), 1034-ERM	1	3 V DC, ERM, Ø10 mm,12,000 rpm	Centrally mounted beneath sample loading slot

Supplementary Table 5 | Comparison of sensor performance in the detection of highly toxic metals.

Biosensor	Detection limit	Linear range of quantification	Response time	Reference
Sphingobium SA2 (pR2GK-merA)	Hg (3 nM)	0-40 nM (R^2=0.95)	5 h	[14]
Escherichia coli (pENTR/D-TOPO-rfp-merR)	Hg (50 nM)	50 nM-10 μM (R^2=0.9848)	≤3 h	[7]
Bacillus megaterium (pRSET/EmGFP)	Cd (0.142 μg L^{-1})	0-10 mg L^{-1} (R^2=0.9647)	4 h	[15]
E. coli (Pmer-merR(m)-Pmer-*luxCDABE*-Δ*cysI*)	Cd (0.005 mg L^{-1})	0.005-2 mg L^{-1} (R^2=0.9921, nonlinear response)	15 min	[16]
E. coli (pT-P-Cd-ABE-Hg-C)	Cd (4.9 nM)<br>Hg (0.5 nM)	Cd (4.9 nM-40 μM)<br>Hg (3.7-468.8 nM)	4 h	[4]
E. coli (pMB1G-Hg-HRV3Cp-SsrA)	Hg (1.4 nM)	Hg (1-100 nM, nonlinear response)	≤4 h	[17]
E. coli (pXW109Hg(RS-E11-RinA)2)	Hg (≤0.01 ppb)	Hg (0.01-2 μg L^{-1}, nonlinear response)	6 h	[18]
V. natriegens (pPcad-mcherry)	Cd^{2+} and Hg^{2+} (10 μg L^{-1})	10-100 μg L^{-1} (R^2=0.9862)	7.5 h	This work

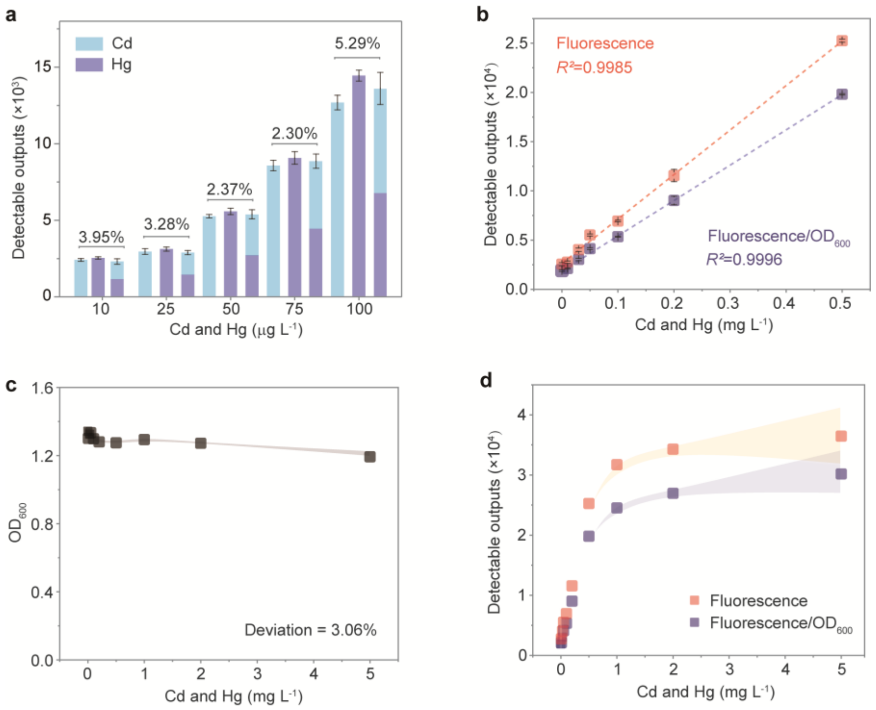

Supplementary Figure 1 | Minimal growth disturbance of the whole-cell biosensor under toxic-metal challenge. a) Detection deviation of the single and collective detection of different concentrations (10–100 μg L^{-1}) of highly toxic metals. **b**) Collective quantification of highly toxic metals with fluorescence and fluorescence/OD_{600} as detectable outputs. Data are presented as mean ± s.d. (n=4). **c**) Growth deviation of whole-cell biosensor exposed to graded concentrations of highly toxic metals. Data are presented as mean ± s.d. (n=4). **d**) Detectable outputs of biosensor on exposure to highly toxic metals. Data are presented as mean ± s.d. (n=4).

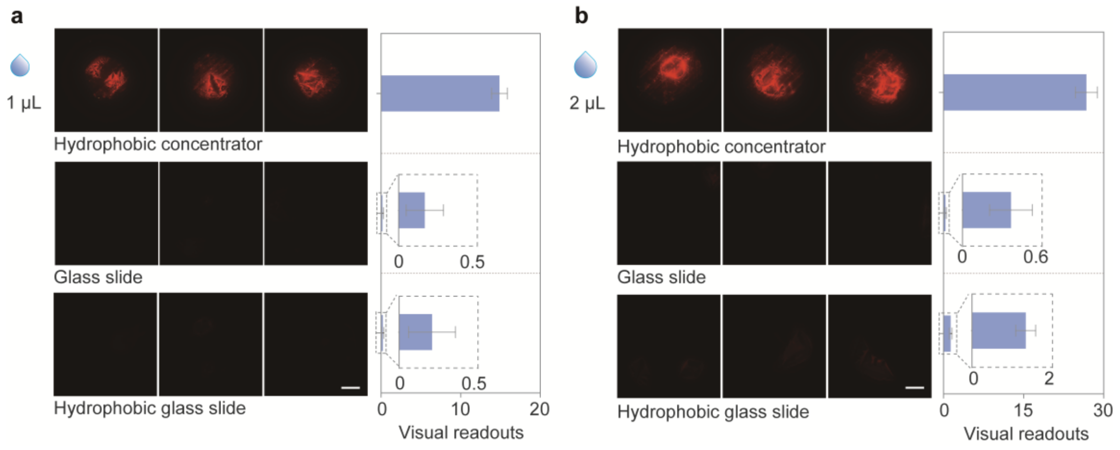

Supplementary Figure 2 | Signal amplification of visual detection by the hydrophobic concentrator. a) 1 µL and **b**) 2 µL microdroplets of identical analytes dropped on different substrates. Fluorescence micrographs (left) were recorded by fluorescence microscope under identical exposure settings with three repeated trials. Scale bar: 500 µm. Corresponding visual readouts (right) were calculated by ImageJ. Data are presented as mean ± s.d. (n=3).

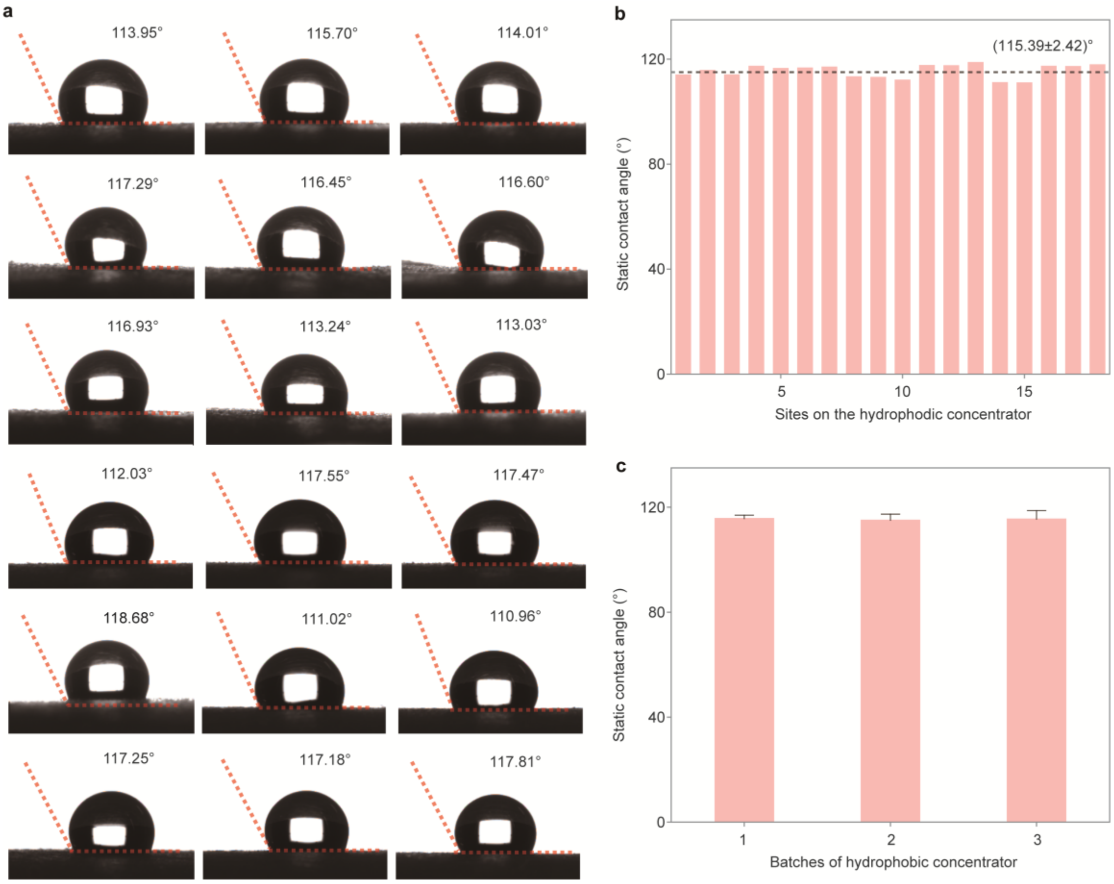

Supplementary Figure 3 | Hydrophobicity uniformity and batch-to-batch reproducibility of the tailor-made hydrophobic concentrator. a) Quantitative assessment of substrate hydrophobicity via static contact angle measurements across 18 random sites on the hydrophobic concentrator. **b**) Uniformity assessment across different locations of the hydrophobic concentrator. **c**) Reproducibility evaluation among three independently fabricated batches. Data are presented as mean ± s.d. (n=6).

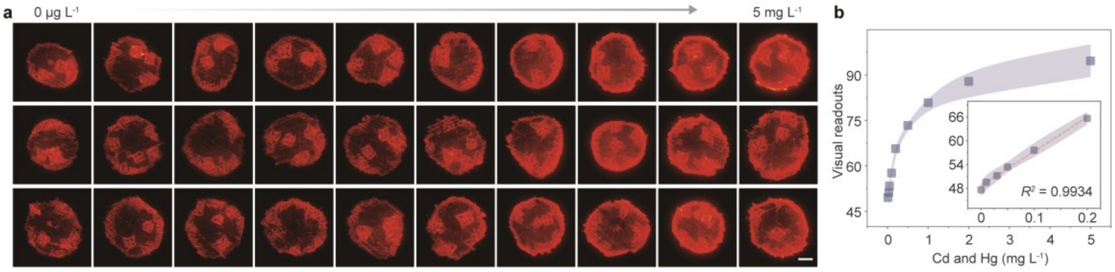

Supplementary Figure 4 | Reproducibility of biosensor-based visual detection on the hydrophobic concentrator. a) Fluorescence micrographs of 3-µL microdroplets containing highly toxic metals (0-5 mg L^{-1}) with three independent replicates at fixed exposure time. Scale bar: 500 µm. **b**) Corresponding visual readouts derived from **a**). Visual readouts were quantified by ImageJ. Data are presented as mean ± s.d. (n=3).

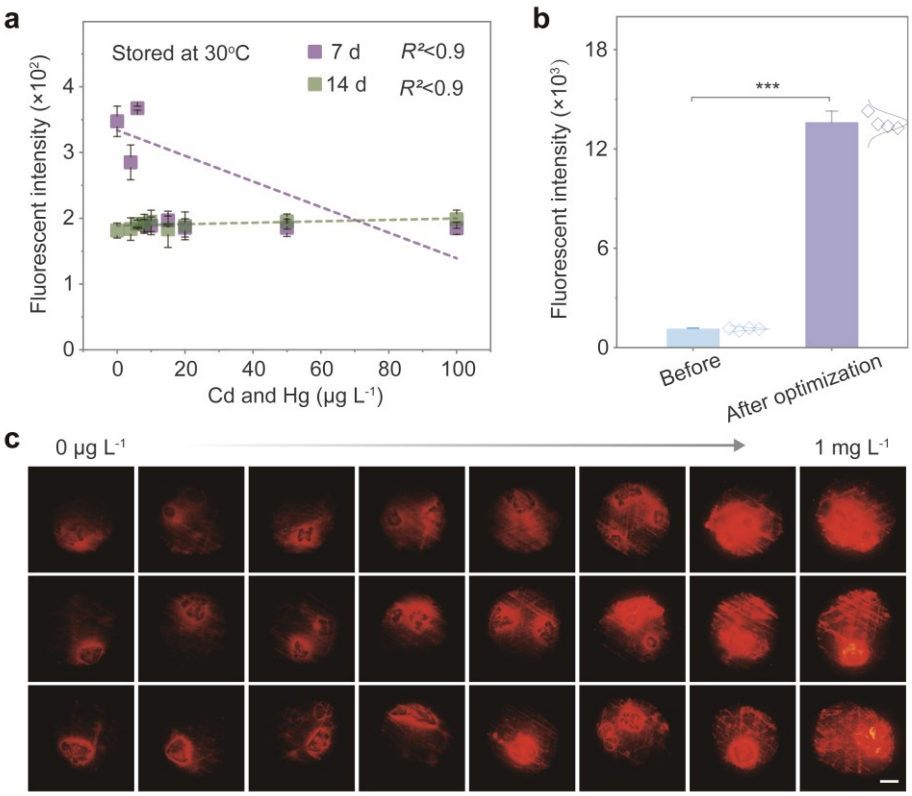

Supplementary Figure 5 | Performance of lyophilized biosensor in storability and detection. a) Stability assessment of lyophilized biosensor after 14 days of storage at 30°C. Data are presented as mean ± s.d. (n=4). **b**) Lyophilization optimization for enhanced fluorescence signals. Data are presented as mean ± s.d. (n=4). *P* value was determined by a two-tailed unpaired t-test. **c**) Fluorescence micrographs of microdroplets (3 μL) containing lyophilized biosensor and mixed toxic metals (Cd^{2+}:Hg^{2+}=1:1) on hydrophobic concentrator. Scale bar: 500 μm. * $P < 0.05$, ** $P < 0.01$, *** $P < 0.001$. ns, not significant ($P \geqslant 0.05$).

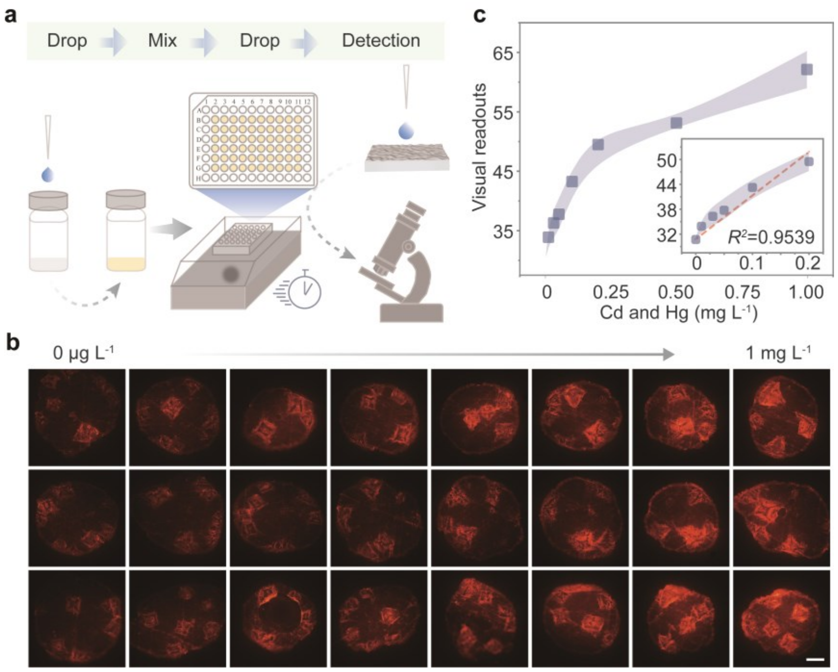

Supplementary Figure 6 | Visual detection of river water spiking highly toxic metals with lyophilized biosensor. **a**) Schematic of visual detection. River water sample mixed with lyophilized biosensor in a mini-oscillator and corresponding microdroplets then applied to the hydrophobic concentrator and imaged using a fluorescence microscope. **b**) Fluorescence micrographs of microdroplets (3 μL) after exposure to river water spiking highly toxic metals at fixed exposure time with three repeated trials. Scale bar: 500 μm. **c**) Dose-response curves derived from **b**). IntDen/Area value as visual readouts from ImageJ analysis. Data are presented as mean ± s.d. (n=3).

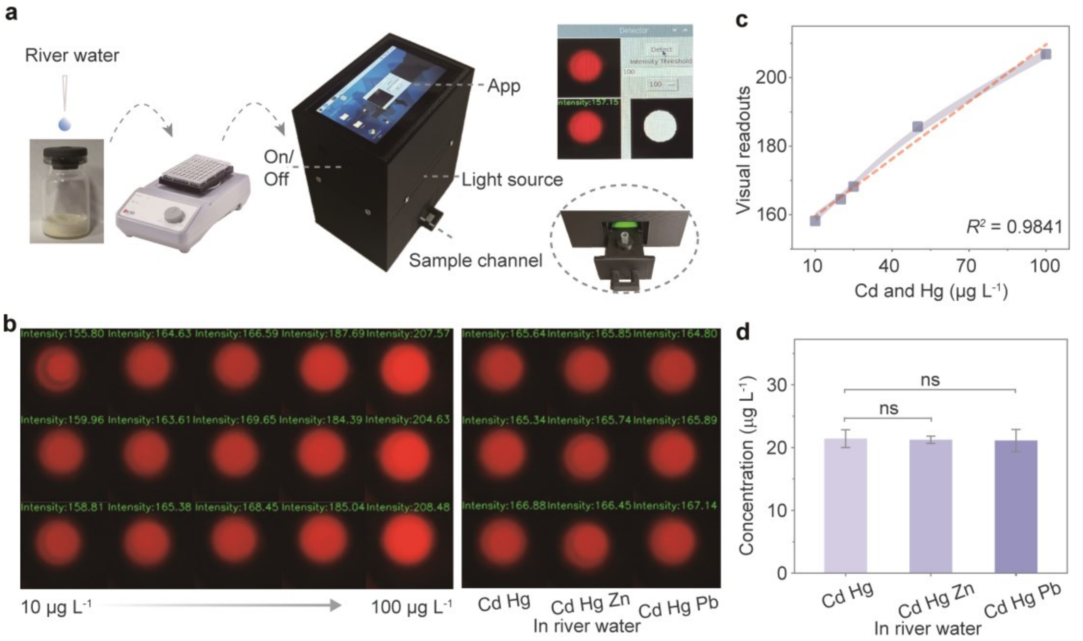

Supplementary Figure 7 | Preliminary detector for portable biosensor quantification of highly toxic metals, employing a commercial mini-oscillator for sample mixing. **a**) Schematic of device and workflow. **b**) Fluorescence images of biosensor after exposure to river water spiked with highly toxic metals (20 μg L^{-1}) in the presence or absence of Zn^{2+} (200 μg L^{-1}) or Pb^{2+} (20 μg L^{-1}) at fixed exposure time with three replicates. **c**) Dose-response curves of fluorescence intensity versus concentration of highly toxic metals derived from **b)**. Data are presented as mean ± s.d. (n=3). **d**) Quantitative results in the presence or absence of Zn^{2+} or Pb^{2+}. Data are presented as mean ± s.d. (n=3). *P* value was determined by a two-tailed unpaired t-test. * $P < 0.05$, ** $P < 0.01$, *** $P < 0.001$. ns, not significant ($P \geqslant 0.05$).

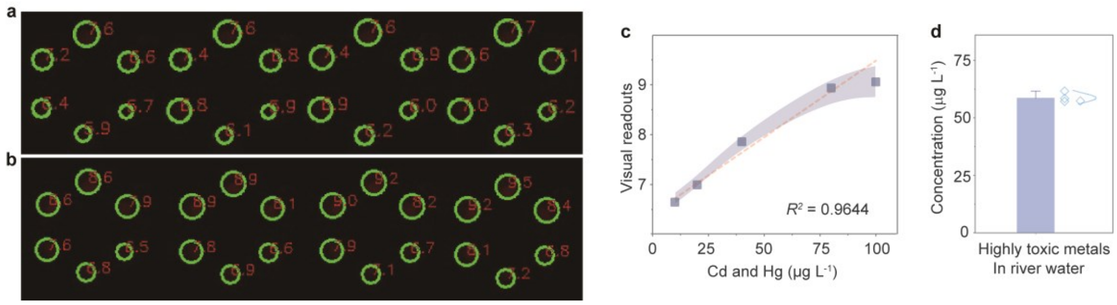

Supplementary Figure 8 | Upgraded detector imaging river water spiked with highly toxic metals. a-b) Raw fluorescence images exhibiting overlaid visual readouts. Each sample was imaged four times within one hour. River samples are real river water spiking with collective Cd^{2+} and Hg^{2+} (50 μg L^{-1} in **a**, and 60 μg L^{-1} in **b**). Note: Original detector-exported image with numerical readout; resolution is low. **c**) Calibration curve (correlates with **b**). Data are presented as mean ± s.d. (n=4). **d**) Validation of detector using river water samples spiked with 60 μg L^{-1} highly toxic metals. Data are presented as mean ± s.d. (n=4).

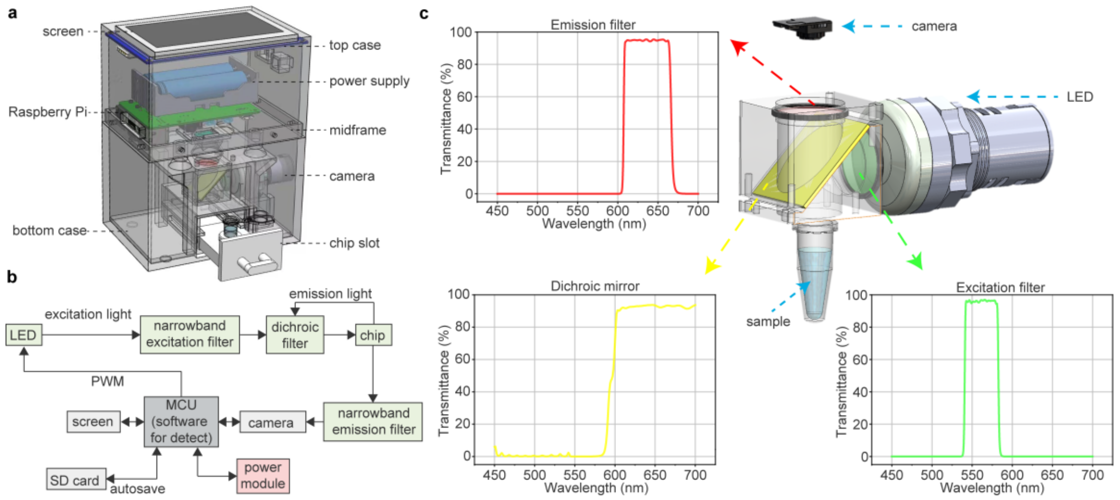

Supplementary Figure 9 | Schematic overview of the primary detector. a) Exploded view of internal components. **b)** Working principle of imaging fluorescent detection. **c)** Compact optical core (excitation filter, dichroic mirror, and emission filter).

REFERENCES

1. Dias, A., Alvarez, M., Gándola, Y., Deisting, A., Posse, E.A., Arnaldi, H., Asorey, H., Bertou, X., Colque, A., Favela-Pérez, F., et al. (2025). PlomBOX: a low cost bioassay for the sensitive detection of lead in drinking water. Commun. Eng. *4*, 1-12.
2. Hu, C.Y., McManus, J., Aghlmand, F., Mei, T., Larsson, E., Emami, A., Murray, R.M. (2025). A portable arsenic sensor integrating *Bacillus megaterium* with CMOS technology. ACS Synth. Biol. *14*, 1615-1624.
3. Aghlmand, F., Hu, C.Y., Sharma, S., Pochana, K., Murray, R.M. (2023). A 65-nm CMOS fluorescence sensor for dynamic monitoring of living cells. IEEE J. Solid-State Circuits *58*, 3003-3019.
4. Hu, S.-Y., Hui, C.-Y., Wu, C., Gao, C.-X., Huang, Z.L., Guo, Y. (2024). Dual-colored bacterial biosensor responsive to cadmium, mercury, and lead for detecting heavy metal pollution in seawater. Ecol. Indic. *166*, 112244.
5. Lopreside, A., Montali, L., Wang, B.J., Tassoni, A., Ferri, M., Calabretta, M.M., Michelini, E. (2021). Orthogonal paper biosensor for mercury(II) combining bioluminescence and colorimetric smartphone detection. Biosens. Bioelectron. *194*, 113569.
6. He, M.-Y., Lin, Y.-J., Kao, Y.-L., Kuo, P., Grauffel, C., Lim, C., Cheng, Y.-S., Chou, H.-H.D. (2021). Sensitive and specificcadmium biosensor developed by reconfiguring metal transport and leveraging natural gene repositories. ACS Sens. *6*, 995-1002.
7. Guo, M.Z., Wang, J.L., Du, R.X., Liu, Y.E., Chi, J.N., He, X.Y., Huang, K.L., Luo, Y.B., Xu, W.T. (2020). A test strip platform based on a whole-cell microbial biosensor for simultaneous on-site detection of total inorganic mercury pollutants in cosmetics without the need for predigestion. Biosens. Bioelectron. *150*, 111899.
8. Buffi, N., Beggah, S., Truffer, F., Geiser, M., Lintel, H.V., Renauda, P., Meer, J.R.V.D. (2016). An automated microreactor for semi-continuous biosensor measurements. Lab Chip *16*, 1383-1392.
9. Truffer, F., Buffi, N., Merulla, D., Beggah, S., Lintel, H.V., Renaud, P., Meer, J.R.V.D., Geiser, M. (2014). Compact portable biosensor for arsenic detection in aqueous samples with *Escherichia coli* bioreporter cells. Rev. Sci. Instrum. *85*, 015120.
10. Jouanneau, S., Durand, M.J., Thouand, G. (2012). Online detection of metals in environmental samples: comparing two concepts of bioluminescent bacterial

biosensors. Environ. Sci. Technol. *46*, 11979-11987.

11. Siegfried, K., Endes, C., Bhuiyan, A.F., Kuppardt, A., Mattusch, J., van der Meer, J.R., Chatzinotas, A., Harms, H. (2012). Field testing of arsenic in groundwater samples of Bangladesh using a test kit based on lyophilized bioreporter bacteria. Environ. Sci. Technol. *46*, 3281-3287.
12. Xu, J.Q., Dong, F., Wu, M.X., Tao, R.S., Yang, J.J., Wu, M.B., Jiang, Y., Yang, S., Yang, L.R. (2021). *Vibrio natriegens* as a pET-compatible expression host complementary to *Escherichia coli*. Front. Microbiol. *12*, 627181.
13. Guo, Y., Hui, C.-Y., Zhang, N.-X., Liu, L., Li, H., Zheng, H.-J. (2021). Development of cadmium multiple-signal biosensing and bioadsorption systems based on artificial *cad* operons. Front. Bioeng. Biotechnol. *9*, 585617.
14. Rayhan Mahbub, K.R., Krishnan, K., Naidu, R., Megharaj, M. (2017). Development of a whole cell biosensor for the detection of inorganic mercury. Environ. Technol. Innov. *8*, 64-70.
15. Rathnayake, I.V.N., Megharaj, M., Naidu, r. (2021). Green fluorescent protein based whole cell bacterial biosensor for the detection of bioavailable heavy metals in soil environment. Environ. Technol. Innov. *23*, 101785.
16. Wei, Y.J., Shi, D.Y., Chen, T.J., Zhou, S.Q., Yang, Z.W., Li, H.B., Yang, D., Li, J.W., Jin, M. (2024). CRISPR/Cas9-based engineered *Escherichia coli* biosensor for sensitive and specific detection of Cd(II) in drinking water. Chemosphere *362*, 142607.
17. Zhang, F.Y., Wang, S.C., Sun, X.T., Li, Z.Q., Fan, X., Mei, M., Yun, Y.L., Yi, L., Zhang, G.M. (2025). Versatile and tunable dual-layer signal amplifier enables ultrasensitive cellular sensors for chemicals. Biosens. Bioelectron. *288*, 117756.
18. Wan, X.Y., Volpetti, F., Petrova, E., French, C., Maerkl, S.J. (2019). Cascaded amplifying circuits enable ultrasensitive cellular sensors for toxic metals. Nat Chem Biol *15*, 540-548.
